# Report on Operative Dentistry

**Published:** 1871-03

**Authors:** Geo. H. Cushing


### ARTICLE III.

### *Report on Operative Dentistry.*

Read before the American Dental Association.

By Geo. H. Cushing, D. D. S.

(*Concluded.*)

It is not claimed in this report for heavy foils that they greatly facilitate operations; on the contrary, in most cases it is thought that more time is required than with light foils; but a far higher claim is made for them, viz: that they bring the conscientious practitioner nearer to the goal for which he is striving—perfection in his operations.

No man who desires to secure the highest results should rest contented with old methods until he has given the heavy foils a thorough and impartial trial.

Before leaving the subject of changes and improvements, it is proper to refer to the methods of performing operations for the removal of salivary calculus, and of polishing the teeth, the importance of which is daily becoming more fully recognized by the profession, and the manner of performing which is receiving a degree of attention more in accordance with its importance than formerly.

A marked advance has clearly been made in this direction both theoretically and practically, and has been materially aided by the improved and delicate instruments which have more recently taken the place of the old fashioned and clumsy forms which were illy adapted for the work; while in certain cases the rubber dam comes in as a most important and indispensible aid to complete thoroughness. In fact progress is the watch-word of operative dentistry in all its details. But the already extreme length of this report precludes any further elaboration of minor points, though a chapter might appropriately be devoted to the operation of pivoting teeth.

Under the head of materials used for filling teeth, only preparations of gold will be considered. Of Watts' crystal gold it is unnecessary to speak; its reputation is fully established, and though having comparatively few advocates, yet it is unquestionably true that very superior and durable operations may be and are made with it.

Brief mention will be made of such preparations of gold as are in any sense new, within the period which this report proposes to cover. The preparation known as "Morgan's Plastic" and "Lamm's Shredded" gold are those most noticeable, as although they are among the things that are past as far as occupying a prominent position before the profession is concerned, they deserve notice here for the brief hour of triumph they enjoyed, and also because in the case of the "plastic" it still fulfills certain indications better perhaps than any thing else.

The first of Morgan's plastic gold which was offered to the profession was better than any since manufactured, and seemed to give promise that better results might be hoped for from its use by the general practitioner than had been previously attained by the use of foil. Experience, however, soon demonstrated that for general use it was not as reliable as foil, while the deterioration in quality, which was soon observed, forced some of its warmest advocates early to abandon its use. The unwise advocacy of some of its earliest friends in the promises held out by them of extraordinary facility and rapidity of operating which might be attained by its use, encouraged a degree of carelessness in its application which was reprehensible and unfortunate to the last degree. The inevitable consequences of this soon became apparent in rapidly disintegrating fillings, and the speedy recurrence of decay at the margins of others which apparently remained intact.

It can hardly be doubted that much more harm than good resulted from the introduction of this form of gold. And yet very fine operations were made with it and can be still. There are in existence operations which were made with it when it was first introduced that will compare very favorably with the best then made with foil. These, however, were obtained as all first class operations are—by thoroughness and care and time—skillfully bestowed. But it can not be doubted that the use of foil secures less disastrous results in general practice than any form of crystal gold.

The plastic gold, however, is in some cases of great advantage still, as in the case of certain cavities whose base is necessarily left concave, and where difficulty is experienced in making secure anchorages by retaining points. In such cases advantage can be taken of the quality of this form of gold which admits of its being packed upon such a surface without rocking, thus enabling the operator to secure a firm base for his filling more easily than could be done with foil; after which the filling can be completed with foil.

Of Lamm's gold it may be said that it never was as popular as Morgan's, and the same general objections to Morgan's apply also to this, and its use was always more limited and is now believed to be exceedingly small.

Of the different qualities of foil there is not very much to be said; very many manufacturers making a superior article, each having his especial advocates among the profession.

Of the light foils mention may properly be made of corarugted foil, manufactured by R. S. Williams, of New York, as being new, in that it was corrugated. For some reason it seemed to work more satisfactorily than the foil of the same manufacturer which was not thus prepared. With many operators this was and is a very favorite style.

Of the heavy foils the same general remarks are true. All first class manufacturers make a good article, and the only make which it would be proper to distinguish from others in this report is the rolled gold.

This unquestionably possesses in a greater degree than any other foil the requisites of a perfectly cohesive foil; that is, softness, pliancy, toughness and cohesiveness. This might naturally be expected in a foil which is not produced by hammering, the latter process depriving gold to a certain extent of the qualities above enumerated. Another style of heavy gold has quite recently been introduced, called "Eureka," made by Mr. Pack. This has not been in use long enough to warrant this report in speaking particularly of its merits, but theoretically it ought to possess the requisite qualities in a high degree, as it is understood to be produced by rolling, though with some modifications of the process as followed by Mr. R. S. Williams, the manufacturer of the rolled gold first alluded to. Whether these modifications tend to improve the quality of the foil is not known, and experience only will determine. It may be remarked in closing this reference to materials, that, properly manipulated, any heavy foils of first class manufacturers will produce the highest results attainable with any form of gold.

Under the head of appliances introduced tending to render operations both more simple and more nearly perfect, it it the duty and pleasure of this report to name as foremost of all such appliances the "rubber dam."

In June, 1864, at a meeting of the New York Dental Society, Dr. J. W. Clowes, in behalf of Dr. S. C. Barnum, of New York, formally presented to the profession as a free gift, this improvement, which had but just previously been devised. It is not too much to say that Dr. Barnum gave to the profession in the "rubber dam" the greatest single invention towards perfecting the operative department of dentistry that has ever been given to it since the commencement of its history. And it is but proper that this report should pay the just tribute of thanks to the author of so valuable an improvement for the manner of its presentation to the profession. It comes without fee or royalty, a gift laid in a true professional spirit upon the altar of science! What higher praise could be spoken ?

It might be deemed inappropriate that such words should be spoken here; but when it is considered how very rarely such an opportunity presents itself for such acknowledgment, the propriety of this public recognition of an act of generosity handsomely performed must be conceded.

The introduction of the rubber dam may be said to have inaugurated a new era in the practice of operative dentistry.

It renders possible the performance of operations which, without its aid, could never be performed. It simplifies operations which under skillful hands were possible before, but only possible under great embarassments, and only to the most skillful in the use of those means which were then known for excluding moisture from cavities to be filled. It simplifies all operations in which it can be applied (and that is in almost every case) by securing immunity from moisture, thus relieving the operator from all anxiety on that score, enabling him to concentrate his attention upon the operation exclusively, and to give all the time necessary for its most thorough performance. In the preparation of many cavities, a free observation of which is obscured by too great a flow of saliva, or by blood in cases where the cavities extend below the margins of the gum, the dam is almost indispensible, and is of essential service where continued applications are required to cavities for the purposes of bleaching or medication.

It is valuable, also, in many cases in the performance of operations for the removal of salivary calculus and of superficial decay. Enabling the operator in the first case to see minute particles which would otherwise escape observation, and in the other case to more thoroughly examine the surfaces of the teeth affected with disease.

Its value is inestimable, and no man who desires to secure the highest results and to most largely benefit his patients, can afford to be without it.

Unfortunately its use is not as universal as it should be, mainly because of the seeming difficulty of its application to beginners. Many are discouraged and do not persevere because they fail to apply it successfully and easily at the first few trials; but no man should rest satisfied until he has mastered the manipulations necessary to its successful application. There is nothing in the details of practice that will so well repay the operator to acquire the knowledge necessary to the proper application of the "rubber dam."

The nearer approach to perfection in the manufacture of instruments, particularly of pluggers, which has been attained quite recently, and the many admirable forms which have been introduced to the profession by various operators during the last few years have contributed materially towards the improved practice of the present day. Of these, the set known as "Varney's pluggers," manufactured by S. S. White, deserve especial mention for their admirable form and the very superior manner of their manufacture and finish.

One of the most admirable inventions tending to lessen and render more perfect labor in this department has been laid before the profession during the past six months, in the shape of a "Pneumatic Burring Engine," the invention of Mr. G. F. Greene, of Kalamazoo, Mich., a gentlemen not of our profession, but an inventor to whom the profession are largely indebted, not only for the instrument itself, but for the demonstration it affords for the practicability of the application of the mechanical forces to instruments to be used upon the teeth. This instrument, as its name indicates, is operated by air, which is drawn through a chamber in which are placed the rotating arms which give motion to the elongated shaft running from this chamber. In the end of this shaft are to be fitted, as required, burrs of various shapes and sizes.

The motion is very rapid and uniform, rendering the instrument entirely manageable. The purpose of this instrument is to lessen the labor of, and secure greater perfection in the operating of burrs and drills, both for opening cavities and for finishing fillings. It works admirably for both these purposes, and the ease and rapidity with which a large filling may be dressed down and the perfection of the operation when effected with this instrument, must be witnessed to be appreciated.

There is also an attachment by means of which drills and burrs may be used in the reverse direction, or at any angle from the main shaft, thus enabling the operator to shape more satisfactorily and easily certain cavities upon the posterior of molar teeth, and to make retaining pits which without some such apparatus conld never be affected. It is believed that this is the first instrument proposing to operate drills and burrs at different angles ever invented, which could be said to be at all of practical value. There is also an attachment for carrying a file which is in some cases of utility. But the great merit of the instrument lies in its admirable operatlon in opening cavities, burring off fillings and removing superficial decay in places otherwise almost inaccessible.

The instrument must take rank as among the most useful which have ever been offered to the profession.

Another instrument working upon an entirely different principle, but aiming to secure the same results, has quite recently been invented by Dr. J. B. Morrison, of St. Louis. It has not been sufficiently tested by the profession generally to have acquired any reputation, but from what the committee has seen of its operation in the crude and imperfect apparatus exhibited, they are inclined to think that it has some points of merit which in a perfectly constructed machine would make it superior to any other yet produced.

In the introduction of the mallet for condensing fillings, a long step in advance was made, and it has steadily been growing into favor, until now it is rare to find an operator who does not use the mallet in some form, either the hand or automatic.

The first mallets introduced were quite light and made of elastic material, either wood, hard rubber or ivory ; but experience has demonstrated the superiority for general use of a heavy non-elastic mallet.

About four years since the leaden mallet, weighing from six to eight ounces, was introdnced to the attention of the profession by Dr. Forbes, of St. Louis, and has been in quite general use at the West for nearly three years past. More recently it has attracted the general attention and is growing more and more into favor daily. Doubless it will soon be universally adopted.

Its great merit consists in its being less objectionable to the patient, while it is easier for the assistant to use, requiring less care and skill to insure an effective blow.

The philosophy of this would seem to be found mainly in the fact that heavier bodies moving at lower velocities, diffuse their force more throughout bodies struck than is the case with lighter bodies moving at high velocity, in which case the blow is struck so quickly, and the force imparted in like manner, that there is not time for it to be diffused to any extent beyond the tooth. The force in this is as it were concentrated upon the tooth and its immediate surroundings. This would seem to be the explanation why the heavy leaden mallet is less disagreeable as a rule than the lighter elastic one.

Of course there are cases in which the light mallet will be preferred, and there are certain cases in which a heavy *soft rubber* mallet will be found much less disagreeable to the patent than any other. Strange as it may seem, the soft rubber mallet condenses the gold very satisfactorily, and for burnishing is much more effective than any other, as a much heavier blow may be struck with it with less discomfort to the patient.

From experiments made regarding the action of different materials as mallets, the soft rubber would seem to possess equal *vis viva* with the lead or lignum vitæ, while the diffusion of force in the case of the rubber closely approximated that of the lead. This again appears very strange in the light of experiments referred to, as the elasticity of materials of a hard texture seeemed clearly to be the chief influence lessening the diffusion of force, while the rubber a more elastic material than wood, shows a greater diffusion of force than lead.

These facts are mentioned as being curious, not with any view to attempt their philosophical solution.

Practically we must learn by experiment in the mouth what is most desirable to nse, and it is confidently affirmed that careful experiment will establish the superiority of the heavy leaden mallet for general use.

With reference to automatic pluggers it may be remarked that most of them operate upon similar principles, and that for certain purposes and cases they are very available, and in fact valuable, but that nothing has yet been perfected in that line which successfully takes the place of the mallet, intelligently and skillfully used by a competent assistant. The nearest approach to this has been made in the "pneumatic mallet," invented by Mr. G. F. Greene, of Kalamazoo; but this is very far from perfect yet.

A pneumatic mallet, invented by Hyde, as supposed, and of which W. H. Jackson & Co., of Ann Arbor, are proprietors, gives promise of a nearer approach of what is desired, but as at present constructed and operated can be said to possess but little merit. It seems unfortunate that its proprietor or inventor is unwilling to take the advice of experienced men in the profession, and endeavor to improve the instrument, for it is believed that within it are folded the elements of success, and that if fully developed the instrument might become a very valuable one, even though it did not entirely supplant the hand mallet.

A want has long been felt by the profession of files of fine quality and proper shapes for the more thorough finishing of fillings and of roughened surfaces of the teeth, where resort has been had to the file either for the purposes of removing superficial decay or of prophylactic treatment, and it would seem proper in this report to refer to the source from which such superior files may be obtained. These files, of a great variety of forms and of very superior quality and fineness of cut, are manufactured by Vautier and by Baumel & Fils, and can be obtained of G. A. Huguenin, 64 Nassau St. New York.

In submitting this report your committee do so with a realizing sense of its many imperfections, but in the belief that the most prominent feature of change or improvement in methods of practice, in materials used and instruments and appliances introduced, tending toward improvement or simplification of practice, have been fairly stated. Of course it would be impossible in such a report, which already exceeds reasonable limits, to follow out in detail all the steps of progress.

The conclusions arrived at in the discussion of subjects must necessarily largely reflect the individual views of the writer and the other members of the committee; but the endeavor has been made in dealing with all these points to reflect the general view of the profession, so far as it could be ascertained in matters of practice, and as to experience regarding methods which are to a large extent occupying debatable ground.

The consideration of the influences tending to modify this department of practice should naturally be treated of in a report of this character, and it was hoped that one member of this committee would have undertaken that task; but such not having been done, your indulgence is asked for the briefest mention only of some of the main points which seem prominent among such forces, that their mention may perchance suggest thought and stimulate to greater activity the already largely progressive spirit of the profession. The influences tending to progress are to be chiefly found in an ambition to excel, a desire for distinction, and a wish for the greater benefit of humanity.

These impulses when controlled by that higher and holier spirit, the possession of which alone would entitle a man to the distinction of being considered truly professional, must eventually result in the highest degree of human attainment.

When, however, these holier ends are subordinate to those of personal ambition and selfish gain, they are materially dwarfed in their possibilities for good. Happily the spirit of unselfishness is growing in the profession, and there is cause for congratulation thus far and greater hope for the future.

Of influences tending to retard improvement, may be mentioned the ignorance of the public touching the mutual relation existing between the profession and themselves, which is most prominently evinced by their unwillingness to recompense justly the highest order of professional attainment, and which finds support and encouragement from the willingness of many members within the ranks of the profession to pander to this spirit, not of illiberality, but rather of unjustness, born of ignorance.

The force of such influence is very great. If "man can not live by bread alone," it is certainly essential that he have bread, and the temptation under the pressure of necessity to accomodate practice to low demands, rather than to try to educate the public to an appreciation of that which alone ought to be done—the very best in all cases—is so great that many men having the possibilities of greatness latent in them, keep those innate powers hidden at the expense of their patrons and of their own higher development.

It may be broadly asserted that no man should remain in the profession one moment after he ceases to command a just or sufficient recompense to enable him to render to his patients the highest professional skill within his power to give. Our motto should be "Excelsior;" but placed above that should be "for the love of God and humanity—a conscientious discharge of our duty." When this high principle controls our profession, we shall see more gigantic strides made in the progess of our science and art, and we may feel that after we are gone from our present field of labor "men will rise up and call us blessed."

